# Supplementary material for: Neuroimaging of headaches in patients with normal neurological examination: protocol for a systematic review
Source: BMJ Open. 2018 Feb 22;8(2):e020190. doi: 10.1136/bmjopen-2017-020190 (PMC5855383; doi:10.1136/bmjopen-2017-020190)
Supplement: Supplementary file 2 [file bmjopen-2017-020190supp002.pdf]

Table : Timeline for the review

Steps of the review process	Duration
Literature search	1 week
Quality appraisal	1 week
Data extraction	1 month
Synthesis	4 weeks
Writing up	2 months
