# Supplementary material for: Neuroimaging of headaches in patients with normal neurological examination: protocol for a systematic review
Source: BMJ Open. 2018 Feb 22;8(2):e020190. doi: 10.1136/bmjopen-2017-020190 (PMC5855383; doi:10.1136/bmjopen-2017-020190)
Supplement: Author's manuscript [file bmjopen-2017-020190.draft_revisions.pdf]

BMJ Open is committed to open peer review. As part of this commitment we make the peer review history of every article we publish publicly available.

When an article is published we post the peer reviewers' comments and the authors' responses online. We also post the versions of the paper that were used during peer review. These are the versions that the peer review comments apply to.

The versions of the paper that follow are the versions that were submitted during the peer review process. They are not the versions of record or the final published versions. They should not be cited or distributed as the published version of this manuscript.

BMJ Open is an open access journal and the full, final, typeset and author-corrected version of record of the manuscript is available on our site with no access controls, subscription charges or pay-per-view fees (http://bmjopen.bmj.com).

If you have any questions on BMJ Open's open peer review process please email editorial.bmjopen@bmj.com

BMJ Open

Neuroimaging for headaches in patients with normal neurologic examination: protocol for a systematic review

Journal:	*BMJ Open*
Manuscript ID	bmjopen-2017-020190
Article Type:	Protocol
Date Submitted by the Author:	19-Oct-2017
Complete List of Authors:	KENTEU, Averik Bernold; Universite Cheikh Anta Diop Faculte de Medecine de Pharmacie et d'Odontologie, Radiology and Medical Imaging<br>Fogang, Yannick; Bafoussam regional Hospital, Bafoussam, Cameroon<br>Nyaga, Ulrich Flore; Universite de Yaounde I Faculte de Medecine et des Sciences Biomedicales<br>Noubiap, Jean Jacques; Groote Schuur Hospital and University of Cape Town, departement of medicine<br>Tatuene, Joseph; Institute of Infection and Global Health, University of Liverpool, United Kingdom, Brain Infections Group; The Walton Centre for Neurology and Neurosurgery, Liverpool, United Kingdom, Department of Neurology
**Primary Subject Heading**:	Neurology
Secondary Subject Heading:	Radiology and imaging, General practice / Family practice, Emergency medicine
Keywords:	Neurology < INTERNAL MEDICINE, RADIOLOGY & IMAGING, GENERAL MEDICINE (see Internal Medicine), ACCIDENT & EMERGENCY MEDICINE

SCHOLARONE™
Manuscripts

For peer review only - http://bmjopen.bmj.com/site/about/guidelines.xhtml

Neuroimaging for headaches in patients with normal neurologic examination: protocol for a systematic review

Bernold Kenteu, MD[1], Yannick F. Fogang, MD[2], Ulrich Flore Nyaga, MD[3], Jean Jacques Noubiap, MD[4], Joseph Kamtchum-Tatuene, MD[5, 6]

1- Department of Radiology and Medical Imaging, Faculté de Médecine, de Pharmacie et d'Ontostomatologie, Université de Cheikh Anta Diop, Dakar, Senegal
2- Head Neuropsychiatry Department, Bafoussam Regional Hospital, Bafoussam, Cameroon
3- Department of Internal Medicine and Specialties, Faculty of Medicine and Biomedical Sciences, University of Yaoundé I, Yaoundé, Cameroon
4- Department of Medicine, Groote Schuur Hospital and University of Cape Town, Cape Town, South Africa
5- Brain Infections Group, Institute of Infection and Global Health, University of Liverpool, Liverpool, UK
6- Department of Neurology, The Walton Centre for Neurology and Neurosurgery, Liverpool, UK

Authors' email addresses: BK: a.bernold@yahoo.fr; YFF: yanfogang@yahoo.fr; UFN: nyagaflore@gmail.com; JJN: noubiapjj@yahoo.fr; JK-T: jkamtchum@yahoo.fr

Correspondance to: Dr. Averik Bernold Dongmo Kenteu. Department of Radiology and Medical Imaging, Faculté de Médecine, de Pharmacie et d'Ontostomatologie; Université de Cheikh Anta Diop, 5005 UCAD, Dakar, Senegal; Email: a.bernold@yahoo.fr; Telephone number: +221 77 476 48 70

Word count: 1422

ABSTRACT

Introduction

Headache disorders (HD) affect people of all ages and races worldwide. With an estimated global prevalence of 50%, HD are among the most frequent neurologic disorders seen in primary care setting and in neurology practice. Because of the relative rarity of secondary HD, patients' neurologic examination is most often unremarkable. However, the fear of a serious underlying cause and prosecutions as well as the will to relieve patients' anxiety lead to an overuse of neuroimaging investigations. This systematic review aims to summarize data on the prevalence of normal anatomic variants (NAV) and incidentals findings (IF) in order to provide updated evidence on the relevance of neuroimaging in patients with headache and normal neurologic examination.

Method and analysis

Studies reporting neuroimaging findings in patients with headache and normal neurologic examination and published before the 30 September 2017 will be identified by searching PubMed/Medline, and EMBASE (Excerpta Medica Database). Relevant unpublished papers and conference proceedings will also be checked. Full texts of eligible studies will then be accessed and data extracted using a standard data extraction sheet. Studies will be assessed for quality and risk of bias. Heterogeneity of studies will be evaluated by the $\chi 2$ test on Cochrane's Q statistic. The prevalence of normal anatomic variants (NAV) and incidentals findings (IF) across studies and in relevant subgroups will be estimated by pooling the study-specific estimates using a random effects meta-analysis. Funnel plot analysis and Egger's test will be done to detect publication bias. The report of this systematic review will be compliant with the MOOSE guidelines.

Ethics and dissemination

The current study is based on published data; ethical approval is, therefore, not required. The final report of this systematic review will be published in a peer-reviewed journal. Furthermore, findings will be presented at conferences and submitted to relevant health authorities.

For peer review only

INTRODUCTION

Headache disorders (HD) affect people of all ages and races worldwide. With an estimated global prevalence of 50%,(1), HD are among the most frequent neurologic disorders seen in primary care setting and in neurology practice. The International Classification of Headache Disorders differentiates between primary headaches which are disorders caused by independent pathomechanisms and secondary headaches which are symptomatic of another condition known to cause the pain. Primary headaches constitute by far the most represented type of headache disorders, with tension type headache and migraine being the most frequent, with a prevalence of 60% and 15% respectively,(2). Despite their benign character, HD are a global public health problem due to the disability and medication overuse they cause and also their cost to the society,(3,4). In England, migraine is responsible for a loss of 25 million days from work or school every year and is associated with an annual cost of about 17 billion dollars in the United States,(5,6). The diagnosis of headache is based on a thorough history taking and a good physical examination seeking to exclude or confirm a secondary cause. Since the most common type of HD are primary headaches, the physical examination will generally be unremarkable and neuroimaging unnecessary,(7). In spite of the relative rarity of secondary HD, the complex presentation of HD frequently raises the fear of serious underlying causes and thus regularly confront physicians with the question of whether or not to perform neuroimaging. The family request, the relief of patient's anxiety and the fear of lawsuit are others reasons for prescribing neuroimaging. These concerns lead to an overuse of neuroimaging and to the frequent discovery of normal anatomic variants (NAV) and incidentals findings (IF) which most often do not explain the patient's pain,(8–10). Several studies conducted in different settings and using different methodological approaches have produced variable estimates of the prevalence of normal and abnormal brain imaging in patients presenting with headache and normal neurological examination. In order to facilitate decision making for clinicians, we undertake this systematic review to summarize these information and provide updated evidence on the relevance (yield, risk-benefit) of neuroimaging in patients with headache and normal neurologic examination.

For peer review only

OBJECTIVE

The objective of this study is to summarize epidemiological data available on the prevalence of NAV and IF on neuroimaging studies performed in patients presenting with headache and normal neurologic examination.

METHODS

Criteria for considering studies for the review

Inclusion criteria

All observational studies reporting neuroimaging findings in patients presenting with headache and normal neurologic examination will be included without date or language restriction.

Exclusion criteria

- Case series with small sample sizes (less than 30 subjects)
- Studies lacking data to compute prevalence and/or explicit method description.
- Duplicates (for studies leading to more than one publication, only the most comprehensive report including the largest sample size will be considered).
- Studies whose full data will not be accessible even after request from authors.

Search strategy for identifying relevant studies

The research strategy will be implemented in two stages

Bibliographic database searches

A comprehensive and exhaustive search on PubMed/MEDLINE, and EMBASE (Excerpta Medica Database) will be conducted to identify all relevant articles reporting neuroimaging findings in patients presenting with headache and normal neurologic examination and published before the 30 September 2017. Both plain language words and medical subheadings (MeSH) will be used. Abstracts of all eligible papers will be reviewed, and full texts of articles will be accessed through PubMed, Google Scholar, HINARI or journals' websites. The detailed search strategy is shown in table 1.

For peer review only

Table 1	Search strategy for pubmed
Search	Search term
#1	"headache" OR "normal neurologic examination" OR "normal neurological examination" OR "normal physical examination"
#2	"normal variant" OR "normal anatomic variant" OR "incidental findings"OR "intracranial lesions" OR "intracranial abnormalities" OR "aneurysm" OR "arachnoid cyst" OR "cerebral vascular malformation" OR "arteriovenous malformation" OR "developmental venous anomaly" OR "cavernoma" OR "dural fistula" OR "empty sella turcica" OR "primary empty sella" OR "gray matter heterotopia" OR "cortical dysplasia" OR "mega cisterna magna" OR "meningioma" OR "pineal cyst" OR "sinusitis" OR "paranasal sinuses" OR "pituitary tumors" OR "radiologically isolated syndrome" OR "Rathke cyst" OR Sagittal sinus venous lake" OR "vein of Galen aneurysm" OR "vestibular schwanomma" OR "acoustic neuroma" OR "Virchow-Robin space" OR "white matter abnormalities" OR "leukoaraiosis" OR "tumor" OR "hydrocephalus" OR "ventricle asymmetry" OR "hydrocephalus" OR "Arnold-Chiari malformation" OR "extra-axial collection" OR "stroke" OR "infarct-like lesion" OR "cortical changes" OR "structural changes" OR "cerebral venous thrombosis" OR "neuroimaging" OR "brain imaging" OR "CT scan" OR "MRI scan"

For peer review only

Searching for other sources

References of all relevant original and review articles will be scrutinized for potential additional data sources, and their full texts will be accessed in a similar way. Conference proceedings will also be checked to identify relevant unpublished data. In case some full-text papers are not accessible via the internet-based sources, authors will be contacted by email to provide reprints and/or related data

Selection of studies for inclusion in the review

Assessment of eligible papers will be independently conducted by two members of the team using an assessment guide to ensure that the selection criteria are consistently applied. Any disagreement will be solved through arbitration by a third assessor.

Assessment of methodological quality and data reporting

The tool developed by Hoy and colleagues will be used to assess the methodological quality of studies included in this review (see online supplementary appendix 1),(11,12). To each item, we will assign a score of 1 (yes) or 0 (no), and will sum scores across items to generate an overall quality score ranging from 0 to 10. According to the overall scores, we will classify studies as having a low (>8), moderate (6–8), or high (≤5) risk of bias.

Data extraction and management

Search results will be compiled using the citation management software, EndNote X7.2.1. A data extraction sheet will be used to collect the following information:

- General information: first author name, year of publication, year of participants' inclusion, country, type of publication, language of publication (full text).
- Study characteristics: study design, setting (hospital, population, emergency department), sample size, mean or median age, age range, proportions of male participants, proportion of acute versus chronic versus recurrent headache, type of neuroimaging used (CT or MRI, without and/or with contrast), power of the MRI magnetic field (0.35, 0.5, 1.5 or 3 Tesla), qualification or the person reading the images (radiologist, neuroradiologist), qualification of the person doing the clinical assessment (general practitioner, emergency physician, neurologist), proportion of HIV positive, proportion of patients with fever, proportion of patients with history of head trauma, criteria used for the clinical diagnosis and classification of headache, proportion of migraines.
- Neuroimaging findings: will be recorded.

Data synthesis including assessment of heterogeneity

For peer review only - http://bmjopen.bmj.com/site/about/guidelines.xhtml

Data will be analyzed using the software STATA (version 13, StataCorp, College Station, TX, USA). Inter-rater agreement for study inclusion and data extraction will be assessed using Cohen's kappa (κ) coefficient,(13). Study-specific estimates will be determined from the point estimate and the appropriate denominators, assuming a binominal distribution. Then, the study-specific estimates will be pooled through a random-effects meta-analysis to obtain an overall summary estimate of the prevalence across studies, after stabilizing the variance of individual studies using the Freeman-Tukey double arc-sine transformation,(14). Heterogeneity will be evaluated by the $\chi 2$ test on Cochrane's Q statistic,(15) which is quantified by I^2 values, assuming that I^2 values of 25%, 50% and 75%, represent low, medium and high heterogeneity respectively,(16). Where substantial heterogeneity will be detected, a subgroup analysis will be performed to detect its possible sources. Visual analysis of funnel plot and Egger's test will be done to detect publication bias,(17). All tests will be two-sided and statistical significance will be defined as $p < 0.05$.

Results reporting and presentation

The resulting systematic review and meta-analysis will follow the MOOSE guidelines for reporting,(18). The study selection process will be summarized using a flow diagram. Reasons for study exclusion will be described. Quantitative data will be presented in summary tables and forest plots where appropriate. The quality scores and risk of bias for each eligible study will be reported.

Ethics and dissemination

This systematic review and meta-analysis will be based on published data. Therefore, ethical approval is not required. The final report of this study, in the form of a scientific paper, will be published in peer-reviewed journals. Findings will be further presented at conferences and submitted to relevant health authorities. We also plan to monitor publications on the topic and to update the review accordingly. This protocol is written in accordance with recommendations from the Preferred Reporting Items for Systematic Review and Meta-Analysis Protocols

2015 statement,(19).

CONCLUSION

For peer review only

This review will provide epidemiologic data on neuroimaging findings in patients presenting with headache and normal neurologic examination. The resulting information will facilitate clinical decision making for clinicians that take care of patients presenting with headache, a highly prevalent symptom affecting 50% of the population,(1) and accounting for 20% of outpatient visits to neurologists,(20).

Some difficulties may arise during the review. First, there might be a great heterogeneity among the studies in terms of participants selection, type of neuroimaging device used, qualification of experts reading the images and list of neuroimaging findings reported. In case of substantial heterogeneity, a narrative synthesis of data will be preferred to a quantitative meta-analysis. Second, the articles selected might not provide a detailed description of the headache features in order to help us to identify potential predictors of abnormal brain imaging during the subgroup analysis.

For peer review only

REFERENCES

1. WHO | Headache disorders [Internet]. WHO. [cited 2017 Oct 8]. Available from: http://www.who.int/mediacentre/factsheets/fs277/en/

2. Stovner LJ, Andree C. Prevalence of headache in Europe: a review for the Eurolight project. J Headache Pain. 2010 Aug;11(4):289–99.

3. Stovner L, Hagen K, Jensen R, Katsarava Z, Lipton R, Scher A, et al. The global burden of headache: a documentation of headache prevalence and disability worldwide. Cephalalgia Int J Headache. 2007 Mar;27(3):193–210.

4. Cha M-J, Moon H-S, Sohn J-H, Kim B-S, Song T-J, Kim J-M, et al. Chronic Daily Headache and Medication Overuse Headache in First-Visit Headache Patients in Korea: A Multicenter Clinic-Based Study. J Clin Neurol Seoul Korea. 2016 Jul;12(3):316–22.

5. Lawrence D. Goldberg MD. The Cost of Migraine and Its Treatment. Am J Manag Care [Internet]. 2005 Jun 15 [cited 2017 Sep 29];11(New Approaches for the Management and Treatment of Migraine 2 Suppl). Available from: http://www.ajmc.com/journals/supplement/2005/2005-06-vol11-n2suppl/jun05-2069ps62-s67/

6. Steiner TJ, Scher AI, Stewart WF, Kolodner K, Liberman J, Lipton RB. The prevalence and disability burden of adult migraine in England and their relationships to age, gender and ethnicity. Cephalalgia Int J Headache. 2003 Sep;23(7):519–27.

7. Bendtsen L, Birk S, Kasch H, Aegidius K, Sørensen PS, Thomsen LL, et al. Reference programme: Diagnosis and treatment of headache disorders and facial pain. Danish Headache Society, 2nd Edition, 2012. J Headache Pain. 2012 Feb;13(Suppl 1):1–29.

8. Schwedt TJ, Guo Y, Rothner AD. “Benign” imaging abnormalities in children and adolescents with headache. Headache. 2006 Mar;46(3):387–98.

9. Valença MM, Valença LPAA, Menezes TL. Computed tomography scan of the head in patients with migraine or tension-type headache. Arq Neuropsiquiatr. 2002 Sep;60(3A):542–7.

10. Evans RW. Diagnostic testing for the evaluation of headaches. Neurol Clin. 1996 Feb;14(1):1–26.

11. Hoy D, Brooks P, Woolf A, Blyth F, March L, Bain C, et al. Assessing risk of bias in prevalence studies: modification of an existing tool and evidence of interrater agreement. J Clin Epidemiol. 2012 Sep;65(9):934–9.

12. file.pdf [Internet]. [cited 2017 Oct 8]. Available from: http://journals.plos.org/plosone/article/file?type=supplementary&id=info:doi/10.1371/journal.pone.0150970.s005

13. Viera AJ, Garrett JM. Understanding interobserver agreement: the kappa statistic. Fam Med. 2005 May;37(5):360–3.

14. Barendregt JJ, Doi SA, Lee YY, Norman RE, Vos T. Meta-analysis of prevalence. J Epidemiol Community Health. 2013 Nov 1;67(11):974–8.

15. Cochran WG. The Combination of Estimates from Different Experiments. Biometrics. 1954 Mar;10(1):101.

16. Higgins JPT, Thompson SG. Quantifying heterogeneity in a meta-analysis. Stat Med. 2002 Jun 15;21(11):1539–58.

17. Egger M, Davey Smith G, Schneider M, Minder C. Bias in meta-analysis detected by a simple, graphical test. BMJ. 1997 Sep 13;315(7109):629–34.

18. Stroup DF, Berlin JA, Morton SC, Olkin I, Williamson GD, Rennie D, et al. Meta-analysis of observational studies in epidemiology: a proposal for reporting. Meta-analysis Of Observational Studies in Epidemiology (MOOSE) group. JAMA. 2000 Apr 19;283(15):2008–12.

19. Moher D, Shamseer L, Clarke M, Ghersi D, Liberati A, Petticrew M, et al. Preferred reporting items for systematic review and meta-analysis protocols (PRISMA-P) 2015 statement. Syst Rev [Internet]. 2015 Jan 1;4(1). Available from: https://www.ncbi.nlm.nih.gov/pmc/articles/PMC4320440/

20. Bashir A, Lipton RB, Ashina S, Ashina M. Migraine and structural changes in the brain: a systematic review and meta-analysis. Neurology. 2013 Oct 1;81(14):1260–8.

DECLARATIONS

Authors’ contributions

For peer review only

Study conception: JK-T, BK, NJJ, YFF, UFN. Manuscript drafting: BK, UFN. Critical revision of manuscript: JK-T, BK, YFF, UFN, NJJ. Final approval of the version to be published: JK-T, BK, YFF, UFN, NJJ

Data sharing

There is no additional unpublished data from this study available.

Funding

This research received no specific grant from any funding agency in the public, commercial or not-for-profit sectors.

Competing interests

The authors have no competing interests to declare.

For peer review only

PRISMA 2009 Checklist

Section/topic	#	Checklist item	Reported on page #
**TITLE**			
Title	1	Identify the report as a systematic review, meta-analysis, or both.	Page 1
**ABSTRACT**			
Structured summary	2	Provide a structured summary including, as applicable: background; objectives; data sources; study eligibility criteria, participants, and interventions; study appraisal and synthesis methods; results; limitations; conclusions and implications of key findings; systematic review registration number.	Page 2
**INTRODUCTION**			
Rationale	3	Describe the rationale for the review in the context of what is already known.	Page 3
Objectives	4	Provide an explicit statement of questions being addressed with reference to participants, interventions, comparisons, outcomes, and study design (PICOS).	Page 4
**METHODS**			
Protocol and registration	5	Indicate if a review protocol exists, if and where it can be accessed (e.g., Web address), and, if available, provide registration information including registration number.	NA
Eligibility criteria	6	Specify study characteristics (e.g., PICOS, length of follow-up) and report characteristics (e.g., years considered, language, publication status) used as criteria for eligibility, giving rationale.	Page 4
Information sources	7	Describe all information sources (e.g., databases with dates of coverage, contact with study authors to identify additional studies) in the search and date last searched.	Page 4,5
Search	8	Present full electronic search strategy for at least one database, including any limits used, such that it could be repeated.	Page 4,5
Study selection	9	State the process for selecting studies (i.e., screening, eligibility, included in systematic review, and, if applicable, included in the meta-analysis).	Page 5,6
Data collection process	10	Describe method of data extraction from reports (e.g., piloted forms, independently, in duplicate) and any processes for obtaining and confirming data from investigators.	Page 6
Data items	11	List and define all variables for which data were sought (e.g., PICOS, funding sources) and any assumptions and simplifications made.	NA
Risk of bias in individual studies	12	Describe methods used for assessing risk of bias of individual studies (including specification of whether this was done at the study or outcome level), and how this information is to be used in any data synthesis.	Page 6,7
Summary measures	13	State the principal summary measures (e.g., risk ratio, difference in means).	NA
Synthesis of results	14	Describe the methods of handling data and combining results of studies, if done, including measures of consistency (e.g., I^2) for each meta-analysis.	NA

For peer review only - http://bmjopen.bmj.com/site/about/guidelines.xhtml

PRISMA 2009 Checklist

Section/topic	#	Checklist item	Reported on page #
Risk of bias across studies	15	Specify any assessment of risk of bias that may affect the cumulative evidence (e.g., publication bias, selective reporting within studies).	NA
Additional analyses	16	Describe methods of additional analyses (e.g., sensitivity or subgroup analyses, meta-regression), if done, indicating which were pre-specified.	NA
**RESULTS**			
Study selection	17	Give numbers of studies screened, assessed for eligibility, and included in the review, with reasons for exclusions at each stage, ideally with a flow diagram.	NA
Study characteristics	18	For each study, present characteristics for which data were extracted (e.g., study size, PICOS, follow-up period) and provide the citations.	NA
Risk of bias within studies	19	Present data on risk of bias of each study and, if available, any outcome level assessment (see item 12).	NA
Results of individual studies	20	For all outcomes considered (benefits or harms), present, for each study: (a) simple summary data for each intervention group (b) effect estimates and confidence intervals, ideally with a forest plot.	NA
Synthesis of results	21	Present results of each meta-analysis done, including confidence intervals and measures of consistency.	NA
Risk of bias across studies	22	Present results of any assessment of risk of bias across studies (see Item 15).	NA
Additional analysis	23	Give results of additional analyses, if done (e.g., sensitivity or subgroup analyses, meta-regression [see Item 16]).	NA
**DISCUSSION**			
Summary of evidence	24	Summarize the main findings including the strength of evidence for each main outcome; consider their relevance to key groups (e.g., healthcare providers, users, and policy makers).	NA
Limitations	25	Discuss limitations at study and outcome level (e.g., risk of bias), and at review-level (e.g., incomplete retrieval of identified research, reporting bias).	NA
Conclusions	26	Provide a general interpretation of the results in the context of other evidence, and implications for future research.	NA
**FUNDING**			
Funding	27	Describe sources of funding for the systematic review and other support (e.g., supply of data); role of funders for the systematic review.	Page 10

NA: Not applicable for this manuscript since it is a Protocol.

From: Moher D, Liberati A, Tetzlaff J, Altman DG, The PRISMA Group (2009). Preferred Reporting Items for Systematic Reviews and Meta-Analyses: The PRISMA Statement. PLoS Med 6(7): e1000097. doi:10.1371/journal.pmed1000097

For peer review only

PRISMA 2009 Checklist

For peer review only

BMJ Open

Neuroimaging of headaches in patients with normal neurologic examination: protocol for a systematic review

Journal:	*BMJ Open*
Manuscript ID	bmjopen-2017-020190.R1
Article Type:	Protocol
Date Submitted by the Author:	20-Dec-2017

Complete List of Authors:	KENTEU, Averik Bernold; Universite Cheikh Anta Diop Faculte de Medecine de Pharmacie et d'Odontologie, Radiology and Medical Imaging<br>Fogang, Yannick; Bafoussam regional Hospital, Bafoussam, Cameroon<br>Nyaga, Ulrich Flore; Universite de Yaounde I Faculte de Medecine et des Sciences Biomedicales<br>Zafack, Joseline; Laval University, Québec City, Québec, Canada, Department of Preventive and Social Medicine<br>Noubiap, Jean Jacques; Groote Schuur Hospital and University of Cape Town, departement of medicine<br>Tatuene, Joseph; Institute of Infection and Global Health, University of Liverpool, United Kingdom, Brain Infections Group; Malawi-Liverpool-Wellcome Trust Clinical Research Programme

**Primary Subject Heading**:	Neurology
Secondary Subject Heading:	Radiology and imaging, General practice / Family practice, Emergency medicine
Keywords:	Neurology < INTERNAL MEDICINE, RADIOLOGY & IMAGING, GENERAL MEDICINE (see Internal Medicine), ACCIDENT & EMERGENCY MEDICINE

SCHOLARONE™
Manuscripts

Neuroimaging of headaches in patients with normal neurologic examination: protocol for a systematic review

Bernold Kenteu, MD[1], Yannick F. Fogang, MD[2], Ulrich Flore Nyaga, MD[3], Joseline Guetsop Zafack[4], Jean Jacques Noubiap, MD[5], Joseph Kamtchum-Tatuene, MD[6, 7]

1- Department of Radiology and Medical Imaging, Faculté de Médecine, de Pharmacie et d'Ontostomatologie, Université de Cheikh Anta Diop, Dakar, Senegal
2- Head Neuropsychiatry Department, Bafoussam Regional Hospital, Bafoussam, Cameroon
3- Department of Internal Medicine and Specialties, Faculty of Medicine and Biomedical Sciences, University of Yaoundé I, Yaoundé, Cameroon
4- Department of Social and Preventive Medicine, Laval University, Quebec, Canada
5- Department of Medicine, Groote Schuur Hospital and University of Cape Town, Cape Town, South Africa
6- Brain Infections Group, Institute of Infection and Global Health, University of Liverpool, Liverpool, UK
7- Malawi-Liverpool-Wellcome Trust Clinical Research Programme, Blantyre, Malawi

Authors' email addresses:

BK: a.bernold@yahoo.fr; YFF: yanfogang@yahoo.fr; UFN: nyagaflore@gmail.com; JGZ: zjoseline@yahoo.fr; JJN: noubiapjj@yahoo.fr; JK-T: jkamtchum@yahoo.fr

Correspondance to: Dr. Averik Bernold Dongmo Kenteu. Department of Radiology and Medical Imaging, Faculté de Médecine, de Pharmacie et d'Ontostomatologie; Université de Cheikh Anta Diop, 5005 UCAD, Dakar, Senegal; Telephone number: +221 77 476 48 70; Email: a.bernold@yahoo.fr

For peer review

Word count: 2721

For peer review only

ABSTRACT

Introduction

Headache disorders (HD) are among the most frequent neurologic disorders seen in neurology practice. Because secondary HD are rare, patients' examination is most often unremarkable. However, the will to relieve patients' anxiety and the fear of prosecutions lead to overuse of neuroimaging thus resulting in the discovery of incidental findings (IF) or normal variants (NV) that can lead to futile or harmful procedures. Knowing the probability of identifying a potentially clinically significant lesion in patients with isolated headache could facilitate decision making and reduce health costs. This review aims determine the prevalence of incidental findings and normal anatomic variants on neuroimaging studies performed in patients presenting with headache and normal neurologic examination.

Method and analysis

Studies reporting neuroimaging findings in patients with headache and normal neurologic examination and published before the 30 September 2017 will be identified by searching PubMed/Medline, and EMBASE (Excerpta Medica Database). Relevant unpublished papers and conference proceedings will also be checked. Full texts of eligible studies will then be accessed and data extracted using a standard data extraction sheet. Studies will be assessed for quality and risk of bias. Heterogeneity of studies will be evaluated by the $\chi 2$ test on Cochrane's Q statistic. The prevalence of normal anatomic variants (NAV) and incidentals findings (IF) across studies and in relevant subgroups will be estimated by pooling the study-specific estimates using a random effects meta-analysis. Visual analysis of funnel plot and Egger's test will be used to detect publication bias. The report of this systematic review will be compliant with the MOOSE guidelines.

Ethics and dissemination

For peer review only

The current study is based on published data; ethical approval is, therefore, not required. The final report of this systematic review will be published in a peer-reviewed journal. Furthermore, findings will be presented at conferences and submitted to relevant health authorities.

Study registration number: CRD42017079714

STRENGTHS AND LIMITATIONS

- To the best of our knowledge, this will be the first systematic review reporting the prevalence of incidental findings and normal variants in patients with normal neurologic examination undergoing neuroimaging for headache.
- We will use robust statistical analyses tools to summarize pool prevalence across studies and this will ensure the reliability of our estimates.
- A major limitation would be the heterogeneity between included studies in terms of availability of advanced neuroimaging equipment (CT and/or MRI), expertise of the clinician performing the neurologic examination and the radiologist interpreting the scans, variability of the imaging protocols.
- Another possible limitation could be the insufficient description of the clinical features of headaches in the selected studies which would limit the scope of our subgroup analyses and our ability to provide practical recommendations for the selection of patients presenting with headache and normal neurologic examination that deserve brain imaging.

INTRODUCTION

For peer review only

Headache disorders (HD) affect people of all ages and races worldwide. With an estimated global prevalence of 50% [1], HD are among the most frequent neurologic disorders seen in primary care setting and in neurology practice. The International Classification of Headache Disorders differentiates between primary headaches which are disorders caused by independent pathomechanisms and secondary headaches which are symptomatic of another condition known to cause the pain. Primary headaches constitute by far the most represented type of headache disorders, tension-type headache and migraine being the most frequent, with a prevalence of 60% and 15% respectively [2]. Despite their benign character, HD are a global public health problem due to the disability and medication overuse they cause and also their cost to the society [3, 4]. In England, migraine is responsible for a loss of 25 million days from work or school every year and is associated with an annual cost of about 17 billion dollars in the United States [5, 6]. The diagnosis of headache is based on a thorough history taking and a good physical examination seeking to exclude or confirm a secondary cause. Since the most common type of HD are primary headaches, the physical examination will generally be unremarkable and neuroimaging unnecessary [7]. In spite of the relative rarity of secondary HD, the complex presentation of HD frequently raises the fear of serious underlying causes and thus regularly confront physicians with the question of whether or not to perform neuroimaging. The family request, the relief of patient's anxiety and the fear of lawsuit are others reasons for prescribing neuroimaging. These concerns lead to an overuse of neuroimaging and to the frequent discovery of normal variants (NV) and incidentals findings (IF) which most often do not explain the patient's pain [8-11].

IF are defined as apparently asymptomatic intracranial abnormalities that were are clinically significant because of their potential to cause symptoms or influence treatment. They can be classified as vascular (silent brain infarct, lacunes, microbleeds, structural vascular abnormalities, white matter hyperintensities) or non-vascular lesions. The latter can be further

For peer review only - http://bmjopen.bmj.com/site/about/guidelines.xhtml

divided into neoplastic lesions (benign and malignant tumors), non-neoplastic lesions (cysts, inflammatory lesions, hydrocephalus, Arnold-Chiari malformations, and extra-axial collections) [12]. NV are defined as anatomical variants that do not have the potential to cause symptoms and do not need any therapeutic intervention (e.g. large cisterna magna, ventricular asymmetry) [12].

Several studies conducted in different settings and using different methodological approaches have produced variable estimates of the prevalence of normal and abnormal brain imaging in patients presenting with headache and normal neurological examination. Because the discovery of an IF or a NV on a brain imaging can sometimes prompt more worries for the patient and lead to futile and even harmful surgical procedures, knowing the probability of identifying a potentially clinically significant lesion (subset of IF) in patients presenting with isolated headache could help to facilitate decision making for clinicians and reduce health care costs by avoiding a number of unnecessary scans.

REVIEW QUESTION

What is the prevalence of incidental findings and normal anatomic variants on neuroimaging studies performed in patients presenting with headache and normal neurologic examination?

METHODS

This review protocol has been prepared according to the 2015 Preferred Reporting Items for Systematic review and Meta-Analysis Protocols (PRISMA-P) guidelines [13]. A PRISMA-P checklist is provided as supplementary appendix 1. The protocol is registered in the PROSPERO International Prospective Register of systematic reviews, registration number

For peer review only

CRD42017079714. The proposed start date for this review is 15th December 2017 and the entire work is expected to be completed in a maximum of 6 months. The timeline for the review is provided as supplementary appendix 2.

Criteria for considering studies for the review

Inclusion criteria

All observational studies reporting neuroimaging findings in patients presenting with headache and normal neurologic examination will be included without date or language restriction.

Exclusion criteria

- Case series with small sample sizes (less than 30 subjects)
- Studies lacking data to compute prevalence and/or explicit method description.
- Duplicates (for studies leading to more than one publication, only the most comprehensive report including the largest sample size will be considered).
- Studies whose full data will not be accessible even after request from authors.

Search strategy for identifying relevant studies

The research strategy will be implemented in two stages

Bibliographic database searches

A comprehensive and exhaustive search on PubMed/MEDLINE, and EMBASE (Excerpta Medica Database) will be conducted to identify all relevant articles reporting neuroimaging findings in patients presenting with headache and normal neurologic examination and published before the 30 September 2017. Both plain language words and medical subheadings

For peer review only

(MeSH) will be used. Abstracts of all eligible papers will be reviewed, and full texts of articles will be accessed through PubMed, Google Scholar, HINARI or journals' websites. The detailed search strategy for PubMed and EMBASE are shown in Table 1 and Table 2, respectively.

Searching for other sources

References of all relevant original and review articles will be scrutinized for potential additional data sources, and their full texts will be accessed in a similar way. Conference proceedings will also be checked to identify relevant unpublished data. In case some full-text papers are not accessible via the internet-based sources, authors will be contacted by email to provide reprints and/or related data. All sources of additional data will be documented and clearly referenced in order to allow verification if necessary.

Selection of studies for inclusion in the review

Titles and abstracts of records identified through literature search will be independently screened for eligibility by two members of the research team (BK and JKT). Full-texts of studies deemed eligible will be retrieved and further assessed for inclusion by the same investigators. Any disagreement will be resolved by discussion and consensus. If the latter is not reached, arbitration will be sought from a third member of the team (YFF). The interrater agreement for the selection of studies will be assessed using a non-weighted Cohen's kappa statistic [14, 15].

Assessment of methodological quality and data reporting

Two independent assessors (JKT and JJN) will use the Risk of Bias Tool for Prevalence Studies (supplementary appendix 3) [16] to evaluate the methodological quality and risk of

For peer review only

bias for each study using the full-text publication. To each item, they will assign a score of 1 (yes) or 0 (no), and will sum scores across items to generate an overall quality score ranging from 0 to 10. According to the overall scores, we will classify studies as having a low (>8), moderate (6–8), or high (≤5) risk of bias. Risk of bias scores will be presented in a table and interrater agreement will be assessed using a weighted Cohen's kappa statistic [17, 18].

Data extraction and management

Search results will be compiled using the citation management software, EndNote X7.2.1. A data extraction sheet will be used to collect the following information:

- General information: first author name, year of publication, year of participants' inclusion, country, type of publication, language of publication (full text).
- Study characteristics: study design, setting (hospital, population, emergency department), sample size, mean or median age, age range, proportions of male participants, proportion of acute versus chronic versus recurrent headache, type of neuroimaging used (CT or MRI, without and/or with contrast), power of the MRI magnetic field (0.35, 0.5, 1.5 or 3 Tesla), qualification or the person reading the images (radiologist, neuroradiologist), qualification of the person doing the clinical assessment (general practitioner, emergency physician, neurologist), proportion of HIV positive, proportion of patients with fever, proportion of patients with history of head trauma, criteria used for the clinical diagnosis and classification of headache, proportion of migraines.
- Neuroimaging findings in patients with normal neurologic examination.

Data synthesis including assessment of heterogeneity

For peer review only

Data will be analyzed using the software STATA (version 13, StataCorp, College Station, TX, USA). Inter-rater agreement for study inclusion and data extraction will be assessed using Cohen's kappa (κ) coefficient [18]. Study-specific estimates will be determined from the point estimate and the appropriate denominators, assuming a binominal distribution. Then, the study-specific estimates will be pooled through a random-effects meta-analysis to obtain an overall summary estimate of the prevalence across studies, after stabilizing the variance of individual studies using the Freeman-Tukey double arc-sine transformation [19]. Heterogeneity will be evaluated by the $\chi 2$ test on Cochrane's Q statistic which is quantified by I^2 values, assuming that I^2 values of 25%, 50% and 75%, represent low, medium and high heterogeneity respectively [20]. Where substantial heterogeneity will be detected, a subgroup analysis will be performed to detect its possible sources. Visual analysis of funnel plot and Egger's test will be done to detect publication bias [21]. All tests will be two-sided and statistical significance will be defined as $p < 0.05$.

Results reporting and presentation

The resulting systematic review and meta-analysis will follow the MOOSE guidelines for reporting [22]. The study selection process will be summarized using a flow diagram. Reasons for study exclusion will be described. Quantitative data will be presented in summary tables and forest plots where appropriate. The quality scores and risk of bias for each eligible study will be reported.

Ethics and dissemination

This systematic review and meta-analysis will be based on data from ethically approved studies. Therefore, ethical approval is not required. The final report of this study, in the form of a scientific paper, will be published in peer-reviewed journals. Findings will be further

For peer review only

presented at conferences and submitted to relevant health authorities. We also plan to monitor publications on the topic and to update the review accordingly.

DECLARATIONS

Authors' contributions

Study conception: BK, UFN, JJN, JK-T. Manuscript drafting: BK, UFN. Critical revision of manuscript: YFF, JGZ, JJN, JK-T. Final approval of the version to be published: BK, YFF, UFN, JGZ, JJN, JK-T. Guarantor of the review: JK-T.

Data sharing

There is no additional unpublished data from this study available.

Funding

This research received no specific grant from any funding agency in the public, commercial or not-for-profit sectors.

Competing interests

The authors have no competing interests to declare.

REFERENCES

1.WHO. Headache disorders: World Health Organization; 2016 [updated April 2016; cited 2017 6th October]. Available from: http://www.who.int/mediacentre/factsheets/fs277/en/.

For peer review only

2.Stovner LJ, Andree C. Prevalence of headache in Europe: a review for the Eurolight project. J Headache Pain 2010;11(4):289-299.

3.Cha MJ, Moon HS, Sohn JH, et al. Chronic Daily Headache and Medication Overuse Headache in First-Visit Headache Patients in Korea: A Multicenter Clinic-Based Study. J Clin Neurol 2016;12(3):316-322.

4.Stovner L, Hagen K, Jensen R, et al. The global burden of headache: a documentation of headache prevalence and disability worldwide. Cephalalgia 2007;27(3):193-210.

5.Goldberg LD. The cost of migraine and its treatment. Am J Manag Care 2005;11(2 Suppl):S62-67.

6.Steiner TJ, Scher AI, Stewart WF, Kolodner K, Liberman J, Lipton RB. The prevalence and disability burden of adult migraine in England and their relationships to age, gender and ethnicity. Cephalalgia 2003;23(7):519-527.

7.Bendtsen L, Birk S, Kasch H, et al. Reference programme: diagnosis and treatment of headache disorders and facial pain. Danish Headache Society, 2nd Edition, 2012. J Headache Pain 2012;13 Suppl 1:S1-29.

8.Schwedt TJ, Guo Y, Rothner AD. "Benign" imaging abnormalities in children and adolescents with headache. Headache 2006;46(3):387-398.

9.Valenca MM, Valenca LP, Menezes TL. Computed tomography scan of the head in patients with migraine or tension-type headache. Arq Neuropsiquiatr 2002;60(3-A):542-547.

10.Evans RW. Incidental Findings and Normal Anatomical Variants on MRI of the Brain in Adults for Primary Headaches. Headache 2017;57(5):780-791.

11.Strauss LD, Cavanaugh BA, Yun ES, Evans RW. Incidental Findings and Normal Anatomical Variants on Brain MRI in Children for Primary Headaches. Headache 2017;57(10):1601-1609.

For peer review only

For peer review only - http://bmjopen.bmj.com/site/about/guidelines.xhtml

12.Morris Z, Whiteley WN, Longstreth WT, Jr., et al. Incidental findings on brain magnetic resonance imaging: systematic review and meta-analysis. BMJ 2009;339:b3016.

13.Moher D, Shamseer L, Clarke M, et al. Preferred reporting items for systematic review and meta-analysis protocols (PRISMA-P) 2015 statement. Syst Rev 2015;4:1.

14.Cohen J. A coefficient of agreement for nominal scales. Educ Psychol Meas 1960;20(1):37-46.

15.Sim J, Wright CC. The kappa statistic in reliability studies: use, interpretation, and sample size requirements. Phys Ther 2005;85(3):257-268.

16.Hoy D, Brooks P, Woolf A, et al. Assessing risk of bias in prevalence studies: modification of an existing tool and evidence of interrater agreement. J Clin Epidemiol 2012;65(9):934-939.

17.Cohen J. Weighted kappa: nominal scale agreement with provision for scaled disagreement or partial credit. Psychol Bull 1968;70(4):213-220.

18.Viera AJ, Garrett JM. Understanding interobserver agreement: the kappa statistic. Fam Med 2005;37(5):360-363.

19.Barendregt JJ, Doi SA, Lee YY, Norman RE, Vos T. Meta-analysis of prevalence. J Epidemiol Community Health 2013;67(11):974-978.

20.Higgins JP, Thompson SG. Quantifying heterogeneity in a meta-analysis. Stat Med 2002;21(11):1539-1558.

21.Egger M, Davey Smith G, Schneider M, Minder C. Bias in meta-analysis detected by a simple, graphical test. BMJ 1997;315(7109):629-634.

22.Stroup DF, Berlin JA, Morton SC, et al. Meta-analysis of observational studies in epidemiology: a proposal for reporting. Meta-analysis Of Observational Studies in Epidemiology (MOOSE) group. JAMA 2000;283(15):2008-2012.

For peer review only

Table 1: Search strategy for PubMed

#1	"headache*"
#2	“neuroimaging” OR “brain imaging” OR “CT scan” OR “MRI scan”
#3	#1AND #2
#4	Restrict [humans]

Table 2: Search strategy for EMBASE

**Database**		**Search strategy**
Embase	#1	'headache*':ti,ab OR 'cephalgia*':ti,ab OR 'cephalalgia*':ti,ab OR 'cranialgia*':ti,ab OR 'head ache*':ti,ab OR 'cephalodynia*':ti,ab OR ' cephalea*':ti,ab OR 'cerebral pain':ti,ab OR 'head pain':ti,ab OR 'eye pain':ti,ab
	#2	'neuroimaging':ti,ab OR 'brain imaging':ti,ab OR 'tomography':ti,ab OR 'mri':ti,ab OR 'magnetic resonance imaging':ti,ab OR 'mr imaging':ti,ab OR 'nmr imaging':ti,ab
	#3	#1AND #2
Restrict to humans	#4	#3 AND 'human'/de
Filter by type of study	#5	#4 AND ('clinical study'/de OR 'clinical trial'/de OR 'cohort analysis'/de OR 'comparative study'/de OR 'controlled clinical trial'/de OR 'controlled study'/de OR 'family study'/de OR 'major clinical study'/de OR 'medical record review'/de OR 'observational study'/de OR 'prospective study'/de OR 'randomized controlled trial'/de OR 'retrospective study'/de OR 'systematic review'/de)

For peer review only

Table : Timeline for the review

Steps of the review process	Duration
Literature search	1 week
Quality appraisal	1 week
Data extraction	1 month
Synthesis	4 weeks
Writing up	2 months

For peer review only

S2 Table. Quality assessment checklist for prevalence studies (adapted from Hoy et al [1])

Name of author(s):<br><br>Year of publication:<br><br>Study title:		
**Risk of bias items**	**Risk of bias levels**	**Points scored**
1. Was the study's target population a close representation of the national population in relation to relevant variables, e.g. age, sex, occupation?	**Yes** (**LOW RISK**): The study's target population was a close representation of the national population.	0
	**No** (**HIGH RISK**): The study's target population was clearly NOT representative of the national population.	1
2. Was the sampling frame a true or close representation of the target population?	**Yes** (**LOW RISK**): The sampling frame was a true or close representation of the target population.	0
	**No** (**HIGH RISK**): The sampling frame was NOT a true or close representation of the target population.	1
3. Was some form of random selection used to select the sample, OR, was a census undertaken?	**Yes** (**LOW RISK**): A census was undertaken, OR, some form of random selection was used to select the sample (e.g. simple random sampling, stratified random sampling, cluster sampling, systematic sampling).	0
	**No** (**HIGH RISK**): A census was NOT undertaken, AND some form of random selection was NOT used to select the sample.	1
4. Was the likelihood of non-response bias minimal?	**Yes** (**LOW RISK**): The response rate for the study was ≥75%, OR, an analysis was performed that showed no significant difference in relevant demographic characteristics between responders and non- responders	0
	**No** (**HIGH RISK**): The response rate was <75%, and if any analysis comparing responders and non-responders was done, it showed a significant difference in relevant demographic characteristics between responders and non-responders	1
5. Were data collected directly from the subjects (as opposed to a proxy)?	**Yes** (**LOW RISK**): All data were collected directly from the subjects.	0
	**No** (**HIGH RISK**): In some instances, data were collected from a proxy.	1
6. Was an acceptable case definition used in the study?	**Yes** (**LOW RISK**): An acceptable case definition was used.	0
	**No** (**HIGH RISK**): An acceptable case definition was NOT used	1
7. Was the study instrument that measured the parameter of interest (e.g. prevalence of low back pain) shown to have reliability and validity (if necessary)?	**Yes** (**LOW RISK**): The study instrument had been shown to have reliability and validity (if this was necessary), e.g. test-re- test, piloting, validation in a previous study, etc.	0
	**No** (**HIGH RISK**): The study instrument had NOT been shown to have reliability or validity (if this was necessary).	1
8. Was the same mode of data collection used for all subjects?	**Yes** (**LOW RISK**): The same mode of data collection was used for all subjects.	0
	**No** (**HIGH RISK**): The same mode of data collection was NOT used for all subjects.	1
9. Were the numerator(s) and denominato r(s) for the parameter of interest appropriate	**Yes** (**LOW RISK**): The paper presented appropriate numerator(s) AND denominator(s) for the parameter of interest (e.g. the prevalence of low back pain).	0
	**No** (**HIGH RISK**): The paper did present numerator(s) AND denominator(s) for the parameter of interest but one or more of these were inappropriate.	1
10. Summary on the overall risk of study bias	**LOW RISK**	0-3
	**MODERATE RISK**	4-6
	**HIGH RISK**	7-9

1. Hoy D, Brooks P, Woolf A, Blyth F, March L, Bain C, et al. Assessing risk of bias in prevalence studies: modification of an existing tool and evidence of interrater agreement. J Clin Epidemiol. 2012;65: 934-939.

For peer review only

PRISMA-P (Preferred Reporting Items for Systematic review and Meta-Analysis Protocols) 2015 checklist: recommended items to address in a systematic review protocol*

Section and topic	Item No	Checklist item	page
**ADMINISTRATIVE INFORMATION**			
Title:			
Identification	1a	Identify the report as a protocol of a systematic review	Page 1
Update	1b	If the protocol is for an update of a previous systematic review, identify as such	
Registration	2	If registered, provide the name of the registry (such as PROSPERO) and registration number	Page 4, 6
Authors:			
Contact	3a	Provide name, institutional affiliation, e-mail address of all protocol authors; provide physical mailing address of corresponding author	Page 1
Contributions	3b	Describe contributions of protocol authors and identify the guarantor of the review	Page 11
Amendments	4	If the protocol represents an amendment of a previously completed or published protocol, identify as such and list changes; otherwise, state plan for documenting important protocol amendments	
Support:			
Sources	5a	Indicate sources of financial or other support for the review	Page 11
Sponsor	5b	Provide name for the review funder and/or sponsor	Page 11
Role of sponsor or funder	5c	Describe roles of funder(s), sponsor(s), and/or institution(s), if any, in developing the protocol	Page 11
**INTRODUCTION**			
Rationale	6	Describe the rationale for the review in the context of what is already known	Page 5, 6
Objectives	7	Provide an explicit statement of the question(s) the review will address with reference to participants, interventions, comparators, and outcomes (PICO)	Page 6
**METHODS**			
Eligibility criteria	8	Specify the study characteristics (such as PICO, study design, setting,	Page 7

For peer review only

		time frame) and report characteristics (such as years considered, language, publication status) to be used as criteria for eligibility for the review	
Information sources	9	Describe all intended information sources (such as electronic databases, contact with study authors, trial registers or other grey literature sources) with planned dates of coverage	Page 7
Search strategy	10	Present draft of search strategy to be used for at least one electronic database, including planned limits, such that it could be repeated	Page 7, 8, 13, 14
Study records:			
Data management	11a	Describe the mechanism(s) that will be used to manage records and data throughout the review	Page 9
Selection process	11b	State the process that will be used for selecting studies (such as two independent reviewers) through each phase of the review (that is, screening, eligibility and inclusion in meta-analysis)	Page 8
Data collection process	11c	Describe planned method of extracting data from reports (such as piloting forms, done independently, in duplicate), any processes for obtaining and confirming data from investigators	Page 8
Data items	12	List and define all variables for which data will be sought (such as PICO items, funding sources), any pre-planned data assumptions and simplifications	Page 9
Outcomes and prioritization	13	List and define all outcomes for which data will be sought, including prioritization of main and additional outcomes, with rationale	Page 9
Risk of bias in individual studies	14	Describe anticipated methods for assessing risk of bias of individual studies, including whether this will be done at the outcome or study level, or both; state how this information will be used in data synthesis	Page 8
Data synthesis	15a	Describe criteria under which study data will be quantitatively synthesised	Page 9, 10
	15b	If data are appropriate for quantitative synthesis, describe planned summary measures, methods of handling data and methods of combining data from studies, including any planned exploration of consistency (such as I^2, Kendall's τ)	Page 9, 10
	15c	Describe any proposed additional analyses (such as sensitivity or subgroup analyses, meta-regression)	Page 9, 10
	15d	If quantitative synthesis is not appropriate, describe the type of summary planned	Page 9, 10
Meta-bias(es)	16	Specify any planned assessment of meta-bias(es) (such as publication	Page 10

For peer review only

		bias across studies, selective reporting within studies)	
Confidence in cumulative evidence	17	Describe how the strength of the body of evidence will be assessed (such as GRADE)	Page 10

*** It is strongly recommended that this checklist be read in conjunction with the PRISMA-P Explanation and Elaboration (cite when available) for important clarification on the items. Amendments to a review protocol should be tracked and dated. The copyright for PRISMA-P (including checklist) is held by the PRISMA-P Group and is distributed under a Creative Commons Attribution Licence 4.0.**

From: Shamseer L, Moher D, Clarke M, Ghersi D, Liberati A, Petticrew M, Shekelle P, Stewart L, PRISMA-P Group. Preferred reporting items for systematic review and meta-analysis protocols (PRISMA-P) 2015: elaboration and explanation. BMJ. 2015 Jan 2;349(jan02 1):g7647.
S

For peer review only
